# A comparative analysis of the effects of containment policies on the epidemiological manifestation of the COVID-19 pandemic across nine European countries

**DOI:** 10.1038/s41598-023-37751-4

**Published:** 2023-07-19

**Authors:** Chiara Podrecca, Enea Parimbelli, Daniele Pala, Cindy Cheng, Luca Messerschmidt, Tim Büthe, Riccardo Bellazzi

**Affiliations:** 1 Department of Electrical, Computer and Biomedical Engineering, University of Pavia, Pavia, Italy; 2 Technical University of Munich, Munich, Germany

**Keywords:** Biomedical engineering, Health policy

## Abstract

The COVID-19 pandemic has been a catastrophic event that has seriously endangered the world’s population. Governments have largely been unprepared to deal with such an unprecedented calamity, partially due to the lack of sufficient or adequately fine-grained data necessary for forecasting the pandemic’s evolution. To fill this gap, researchers worldwide have been collecting data about different aspects of COVID-19’s evolution and government responses to them so as to provide the foundation for informative models and tools that can be used to mitigate the current pandemic and possibly prevent future ones. Indeed, since the early stages of the pandemic, a number of research initiatives were launched with this goal, including the PERISCOPE (Pan-European Response to the ImpactS of COVID-19 and future Pandemics and Epidemics) Project, funded by the European Commission. PERISCOPE aims to investigate the broad socio-economic and behavioral impacts of the COVID-19 pandemic, with the goal of making Europe more resilient and prepared for future large-scale risks. The purpose of this study, carried out as part of the PERISCOPE project, is to provide a first European-level analysis of the effect of government policies on the spread of the virus. To do so, we assessed the relationship between a novel index, the Policy Intensity Index, and four epidemiological variables collected by the European Centre for Disease Control and Prevention, and then applied a comprehensive Pan-European population model based on Multilevel Vector Autoregression. This model aims at identifying effects that are common to some European countries while treating country-specific policies as covariates, explaining the different evolution of the pandemic in nine selected countries due to data availability: Spain, France, Netherlands, Latvia, Slovenia, Greece, Ireland, Cyprus, Estonia. Results show that specific policies’ effectiveness tend to vary consistently within the different countries, although in general policies related to Health Monitoring and Health Resources are the most effective for all countries.

## The effects of the pandemic

From the beginning, the spread of the SARS-CoV-2 virus has not only had a devastating impact on public health, but also has engendered staggeringly numerous and diverse socio-political responses. Healthcare systems were overwhelmed with treating COVID-19 patients along with their regular patient load not only because of the deadliness of the disease, but in no small part due to insufficient availability of resources (e.g. hospital beds, healthcare professionals), especially intensive care units. Unprecedented disruptions to the global economy occurred not only because of a labor force mowed down by the severity of the disease, but also because of policies that, in seeking to restrict its spread, also impeded labor practices and supply chains from the local to the global level. Governments clearly play an important role in shaping the evolution of the pandemic, as they have the prerogative to direct medical, social or economic resources. But governments worldwide initially were forced to make monumental decisions in an information-poor environment. Relying on data from previous epidemics could only be of superficial help given qualitative differences in historical periods as well as the differences in virus characteristics. And, because of the rapidity with which the SARS-CoV-2 virus spread, it was especially difficult for governments and policymakers to define “data-driven” strategies for public policy-making. Thanks to the extraordinary efforts carried on worldwide to collect data on the COVID-19 pandemic, it is now possible and of paramount importance to learn from this newly available data in order to contain the effects of the current and future pandemics. To illustrate the powerful potential of such evidence-based public policy-making when the requisite data can be gathered, we present in this article a rigorous analysis of the relationship between government policies and COVID-19 health outcomes, using a trove of new data on government policies (summarised in *Policy Intensity Indices*) and indicators that describe the evolution of the pandemic throughout the year 2020. To do so, we estimate models that can account for potential endogeneity in this relationship since theoretically, epidemiological variables can not only affect the decisions of policymakers, but government policy can also clearly—either positively or negatively—affect the evolution of the pandemic itself.

### The PERISCOPE project

The Pan-European Response to the ImpactS of COVID-19 and future Pandemics and Epidemics (PERISCOPE) Project^1^, was funded by the European Commission’s Horizon 2020 program to undertake multi- and interdisciplinary social science research on the COVID-19 disease. It seeks to support policymakers in the management of the current global pandemic and to prepare Europe for future pandemics and epidemics by providing integrated data, statistical models and policy-making guidance. PERISCOPE researchers have carried out theoretical, experimental and observational-empirical research to contribute to a deeper understanding of the short and long-term impacts of the pandemic and the measures adopted to contain it. A core component of this project is the ongoing effort to build a comprehensive COVID-19 Atlas^2^ to inform users about the dynamics, effects, and consequences of the COVID-19 outbreak. The ATLAS has a dual focus: visual analytics to illustrate the results of predictive models and data analysis, and a WebGIS for geographical exploration of data. A number of dimensions are covered, including data on health systems, socio-economic impacts, mental health and inequalities, health policies, behavioral science, governance, education.

PERISCOPE relies on a multidisciplinary consortium of experts to thoroughly address its challenging objectives. The project leader at the University of Pavia, Italy, coordinates 31 project partners, including academic and other research institutions located throughout Europe.

### Objective

The objective of this article is to improve our understanding of the relationship between Non-Pharmaceutical Interventions (NPIs) during the pandemic (measured through *Policy Intensity Indices*) and epidemiological outcomes in such a way that it allows the epidemiological variables to affect policy, as well as public policies to affect the evolution of the pandemic.

Our objective is also, by studying COVID-19 restrictions, to understand which ones should be prioritized to limit the severity of any pandemic that exhibits similar characteristics to the COVID-19 pandemic. Ultimately, we seek to understand which NPIs are most promising for minimizing the spread of the virus.

The analysis focuses on 9 European countries: Spain, France, Netherlands, Latvia, Slovenia, Greece, Ireland and Cyprus. We focus on the year 2020 time period in order to exclude some known co-factors such as the introduction of vaccines in 2021 and the evolution of COVID-19 variants, since the first Variant Of Concern (VOC) has been classified in December 29th, 2020^3^. The early phase of the pandemic, moreover, is of peculiar interest since political decisions were made hastily.

## Related work

The SARS-CoV-2 virus has attracted significant scholarly attention from a myriad of fields since the beginning of the pandemic. Amid this abundance of academic work, among the debated topics keeping busy researchers has been the relationship between non-pharmaceutical interventions (NPIs), such as lockdowns and travel bans and the spread of COVID-19 in Europe. Early work on the subject has been able to have an outsized influence on our understanding of this relatively new research topic and debate around their findings have helped researchers identify potential weaknesses in research design or data quality for subsequent work to build on. For example, Flaxman et al.^4^, study the effect of major interventions across 11 European countries from the start of the COVID-19 epidemics in February 2020 until 4 May 2020, when lockdowns started to get lifted. They do so by measuring the effect of NPIs on the temporal trend of the $R_T$ score. $R_T$ is an epidemiological quantity that represents the average number of infections generated at time *t* by each infected case In^4^, the $R_T$ score is derived from the reported deaths, which are considered more reliable than reported cases. They find that policy interventions adopted during those first months of the pandemic were sufficient to drive $R_T$ below 1 and achieve control of the pandemic. They arrive at this conclusion by comparing the situation at the time to an hypothetical scenario in which $R_T$ remains constant from the beginning of the pandemic, using a hierarchical Bayesian model.

Flaxman et al.^4^ has come under scrutiny by others in the field. For instance, Bryant and Elofsson^5^ critiqued the paper’s claim that lockdowns accounted for 81% of the reduction in $R_T$, pointing out that though Sweden did not implement any lockdowns, they still experienced a similar decrease in $R_T$.

Soltesz et al.^6^ offered a similar critique of Flaxman et al.’s^4^ findings. Using simulations based on Flaxman et al.’s^4^ original model code, Soltesz et al.’s^6^ analysis suggests that, contrary to^4^ assertions, the effectiveness of individual NPIs cannot be reliably quantified. According to^6^, a major defect in the Flaxman et al’s analysis is that the data on COVID-19 deaths are not sufficiently representative of the disease’s spread to support their conclusions. Moreover, in their simulation analysis, they found that subtle changes in the definitions of NPIs result in a great deal of variation in the estimated effectiveness of the NPIs categories considered. In particular, Soltesz pointed out that the Bayesian model used by Flaxman appears to be sensitive to small perturbations. As such, they argue that it is likely that lockdowns seem to have such high importance in Flaxman et al’s model because they were implemented early compared to other interventions, rather than because they had a real substantial impact.

In response to such critiques, a follow-up article from Flaxman^7^ highlighted that the goal of the original paper^4^ was to examine multiple countries to see what worked in most places, not to explain the trajectory of the epidemic in each individual country, and that the effectiveness of NPIs can in principle be identified when looking at what worked in most countries, subject to the availability of data. Given that more data had become available, and it was evident that there was more heterogeneity across countries, Flaxman^7^ used an extended model, including a random effect, with the aim of capturing country-specific variation in the effectiveness of the government-mandated interventions.

In^8^, Hsiang et al. compiled data on 1700 local, regional. and national non-pharmaceutical interventions that were deployed in the ongoing pandemic across localities in China, South Korea, Italy, Iran, France and the United States. They applied reduced-form econometric methods to empirically evaluate the effect that these anti-contagion policies have had on the growth rate of infections.

Haug et al.^9^ studied as well the effectiveness of NPIs to mitigate the spread of SARS-CoV-2. They quantified the impact of 6,068 4-level hierarchically coded NPIs implemented in 79 territories on the effective reproduction number, $R_t$, of COVID-19, in the period of March-April 2020. The impact of government interventions on $R_t$ is analyzed using harmonized results from a multi-method approach consisting of a case-control analysis, a step function approach to LASSO time-series regression, random forests and transformers.

Brauner et al.^10^ amassed and curated data from 41 countries as input to a model to identify the individual non-pharmaceutical interventions that were the most effective at curtailing transmission during the early pandemic. A data-driven approach was used to estimate the effects that seven NPIs had on COVID-19 transmission in 41 countries between January and the end of May 2020, using a model inspired by Flaxman et al. in^4^, with several additions. Several NPIs were associated with a clear reduction in $R_t$.

Imai et al. in 2020^11^ examined the existing literature, and collated data, on implementation of NPIs to examine their effects on the COVID-19 pandemic. They found out that measures such as travel restrictions have been implemented in multiple countries and appears to have slowed the geographic spread of COVID-19 and reduced initial case number. Furthermore, due to the relatively sparse information on the differences with and without interventions, it is difficult to quantitatively assess the efficacy of many interventions. They concluded that the timely implementation of control measures is key to their success and must strike a balance between early enough application to reduce the peak of the epidemic and ensuring that they can be feasibly maintained for an appropriate duration. Such measures can have large societal impacts and they need to be appropriately justified to the population. As the pandemic of COVID-19 progresses, quantifying the impact of interventions is a vital consideration for the appropriate use of mitigation strategies.

Davies et al. in 2020^12^ performed a modelling study on effects of NPIs on COVID-19 cases, deaths and demand for hospital services. They used a stochastic age-structured transmission model to explore a range of intervention scenarios, in England, Wales, Scotland, and Northern Ireland. The four base interventions modelled were school closures, physical distancing, shielding of people aged 70 years or older, and self-isolation of symptomatic cases. They also modelled the combination of these interventions, as well as a programme of intensive interventions with phased lockdown-type restrictions that substantially limited contacts outside of the home for repeated periods. They found that the four base interventions were each likely to decrease R0, but not sufficiently to prevent ICU demand from exceeding health service capacity. The combined intervention was more effective at reducing R0, but only lockdown periods were sufficient to bring R0 near or below 1; the most stringent lockdown scenario resulted in a projected 120,000 cases (4000–700,000) and 50,000 deaths (9300–160,000). Intensive interventions with lockdown periods would need to be in place for a large proportion of the coming year to prevent health-care demand exceeding availability.

Gokmen et al.^13^ performed a cross-country study with the same aim of investigating the effectiveness of NPIs implemented to control the COVID-19 pandemic. To this end, eight NPI measures were analysed, and their effects on the number of cases were investigated for France, Spain, China, and South Korea. In the study, the treatment effect of these mechanisms on the daily increase rate of the total number of cases during a certain period was analysed by using logarithmic linear regression with a dummy variables model. The findings indicate that the measures are effective against the spread of the pandemic at different levels. The findings also suggest that the most effective measure in decreasing the number of cases is workplace closure. An analysis comparing the effectiveness of countrywide measures and regional measures shows that school closing is the most effective measure to decrease the number of cases when implemented countrywide as opposed to regional implementation.

In another study, Ferguson in 2020^14^ presents the epidemiological model which has informed policymaking in the UK, that is a confront between suppression and mitigation of NPIs interventions. Mitigation focuses on slowing but not necessarily stopping epidemic spread, not to interrupt transmission completely, but to reduce the health impact of an epidemic, while suppression has the aim to reducing the reproduction number, R, to below 1 and hence to reduce case numbers to low levels or (as for SARS or Ebola) eliminate human-to-human transmission. They find that optimal mitigation policies (combining home isolation of suspect cases, home quarantine of those living in the same household as suspect cases, and social distancing of the elderly and others at most risk of severe disease) might reduce peak healthcare demand by 2/3 and deaths by half. However, the resulting mitigated epidemic would still likely result in hundreds of thousands of deaths and health systems (most notably intensive care units) being overwhelmed many times over. For countries able to achieve it, this leaves suppression as the preferred policy option.

Studying the effect on NPIs on the epidemic in European countries, our research introduces several innovations that differentiate it from previous studies. First, since we have the benefit of conducting this study in early 2022, we can avail ourselves of more comprehensive data that covers the whole year of 2020 instead of only a few months. Second, to quantify NPIs, we use a novel measure (defined in the “Data sources” section), the *Policy Intensity Indices*, which allows us to better summarize how active governments were in different policy areas in response to the pandemic. Third, to our knowledge, we are the first to apply a population autoregressive model that is able to derive cross-country conclusions while properly modelling country-specific effects.

## Methods

### Data sources

Our analysis is based on novel measures developed by Kubinec et al.^15^ called *Policy Intensity Indices* (PIIs), which quantify the investment of government responses to COVID-19 within distinct policy areas, and can be defined as a measure of governments’ commitment to a given policy area, e.g. political, social, financial, relatively to other governments. The *Policy Intensity Indices* range from 0 (minimum intensity) to 100 (maximum intensity), with 0 denoting relatively low commitment and 100 denoting high commitment. These daily indices track the changes in policy makers investment in each of the following policy areas from 1 January to the end of 2020, for over 180 countries worldwide: Social Distancing (SD), School Restrictions (SR), Business Restrictions (BR), Health Monitoring (HM), Health Resources (HR), Mask Policies (MP).

The PIIs are created by combining data from two of the most comprehensive COVID-19 datasets: the CoronaNet COVID-19 Government Response Event Dataset^16,17^ and the Oxford COVID-19 Government Response Tracker^18^. Using PIIs allows to quantitatively investigate the role of policy-making activities in preventing infections in the early pandemic period. Since the start of the COVID-19 pandemic, research groups all around the world have invested enormous time and effort into collecting data on government policies that have supposedly sought to prevent the transmission of the virus. CoronaNet in particular has brought many of these efforts together, compiling data from numerous sources under one roof (for EU countries with the support of PERISCOPE). As a consequence, we now have much richer, more fine-grained, and quite comprehensive data on the wealth of different NPIs through which governments have tried to stem the spread of COVID-19. The analysis of highly differentiated measures, however, is often problematic, because distinct but closely related policies are often functional substitutes. For instance, a policy requiring people to wear masks whenever they are in close proximity to others outside of the home differs, strictly speaking, from a policy requiring everyone to wear masks in public spaces, but they aim to achieve the same result through very similar means. Analyses that treat these two policies as unrelated to each other to assess the effectiveness of each one, may falsely conclude that none of them are effective. The indices used in this paper keep major policy areas separate but provide aggregated measures of those distinct policy areas to be able to provide better answers to questions about the effect of disparate policies. They enable us to examine statistically the effect of those policies on the spread of COVID-19 infections, as well as an array of socio-economic outcomes, including mental health.

These indices create such an aggregated measure through a Bayesian measurement model and in doing so allows us to provide a way to account for situations in which multiple, discrete policies shared the same policy goal.

Our second main source of data is the European Centre for Disease Control and Prevention (ECDC) data repository^19^. The ECDC has been monitoring the progression of COVID-19 in Europe since the first outbreaks in 2020. Specifically, it provides weekly reports on number of cases, case/test ratio, cumulative cases, incidence, hospitalizations and mortality in all of Europe and in specific countries. Each report specifies the changes that have been observed in the various countries in the previous week regarding all the aforementioned parameters and summarizes the situation in each country. We used two specific ECDC datasets for our analysis: data on weekly rate of new COVID-19 cases and deaths (per 100,000 population) and data on weekly hospital and ICU admission rates (per 100,000 population), both by date and country (https://www.ecdc.europa.eu/en/ publications-data/data-national-14-day-notification-rate-covid-19; https://www.ecdc.europa.eu/en/publications-data/download-data-hospital-and-icu-admission-rates-and-current-occupancy-covid-19).

### Data preprocessing

In order to exclude potential changes in the relations between the indices and the variables after the introduction of the vaccination campaign and the emergence of new COVID-19 variants, we selected only data for the year 2020 (we used MATLAB R2021b for data preprocessing and preliminary analyses).

While the indices are available with daily observations, the ECDC’s datasets provide only weekly data. For the combined datasets we therefore selected the calendar week as our unit of analysis, obtaining values for the 53 weeks of the year 2020 by aggregating the corresponding up-to-7 daily values of the *Policy Intensity Indices*. Working at the weekly level of temporal granularity also has the benefit of avoiding the noise of daily data, which often contain more inaccuracies and greater variability (e.g., because the cases counts depend on the number and type of tests performed on a given day), compared to weekly data.

Given our interest in the relationship between policy interventions and the epidemiological outcomes, we had to restrict our occupancy, for which data was also contained in the PIIs database. Moreover, most of these 29 countries, had completely missing data for the hospital admission variable and/or the ICU occupancy variable. Only five countries had complete data for the year 2020 for all the variables we wanted to include in our analyses. Four additional countries had an acceptable proportion of missing data (see next section for details).

#### Missing data imputation

Missing data was found to be a substantial issue when preparing our subsequent analyses. We tackled this problem with imputation. In our initial representation, each country had its own dataset, with as many rows as the weeks considered and as many columns as there are epidemiological variables (for instance Table 1 shows the first rows of the France dataset):

**Table 1:** Examples of columns for the first 5 weeks of the France database.

France database				
N week	New cases	admHosp	admICU	Deaths
1	0.180	0.000	0.000	0.001
2	1.457	0.000	0.000	0.253
3	6.382	1.722	0.429	0.160
4	16.735	11.429	2.568	0.813
5	34.886	25.255	5.564	2.870
.	.	.	.	.

- New weekly cases incidence per 100k people (*New cases*).
- Weekly Hospital admission rate per 100k people (*admHosp*).
- Weekly ICU admission rate per 100k people (*admICU*).
- Weekly deaths incidence per 100k people (*Deaths*).

**Figure 1:**
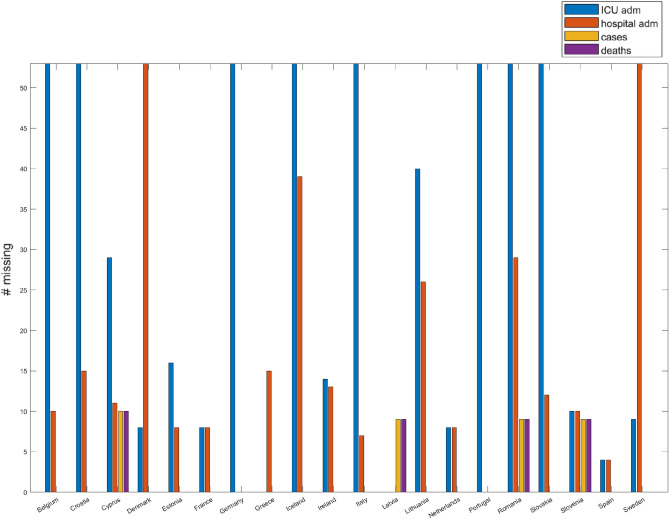
Missingness pattern for the four epidemiological variables when integrating the PIIs and ECDC datasets. For instance, Belgium is completely missing *admICU* variable, as well as Croatia, Germany, Iceland, Italy, Portugal, Romania and Slovakia. Denmark and Sweden are completely missing *admHosp* variable.

Incidence for *New cases* and *Deaths* has been computed dividing the number of new weekly cases (and deaths as well) by the corresponding size of the country population^19^ and calculating the proportion with 100,000 inhabitants. Five countries had mostly complete data for the 53 weeks of the year 2020, with weekly observations for all the four considered variables: Spain (49 complete contiguous observations), France (45 complete contiguous observations), Netherlands (45 complete contiguous observations), Latvia (44 complete contiguous observations), Slovenia (43 complete contiguous observations). Figure 1 shows a bar plot with the missingness pattern found in the four epidemiological variables for the common PIIs and ECDC countries.

All the other countries presented substantial missing data, with some hospitalization variables completely missing. We only considered countries that had data for all the four variables. We ended up with four additional countries, that showed at most $\frac{1}{3}$ of missing data for any of the four variables. The countries included in the dataset were: Greece (45 observations, of which 7 missing values for *admHosp* variable), Ireland (40 observations, of which 1 missing value for the *admICU* variable), Cyprus (25 observations, of which 8 missing values for the *admICU* variables), Estonia (43 observations, of which 6 missing values for the *admICU* variable). The imputation was limited to the weeks between the first and last data point for each country. At the end of this process, we had a total of nine countries for our analyses: Spain (49 weeks), France (45 weeks), Netherlands (45 weeks), Latvia (44 weeks), Slovenia (43 weeks), Greece (45 weeks), Ireland (40 weeks), Cyprus (25 weeks) and Estonia (43 weeks).

### Data analysis

Here we present the overall data analysis process followed in our work. First a simple correlation analysis was performed, both in the form of raw correlation, and cross-correlation in order to account for a temporal lag between the time-series analyzed. Second, Granger causality tests are performed to investigate if the direction in causality relationships between variables—i.e. if PIIs cause the epidemiological variables or vice-versa. Finally, we apply a Population Modelling Approach to discern cross-country from country-specific effects.

#### Correlation analysis

Correlation analyses measure the strength and direction of the linear relationship between two or more variables. They are useful for conducting prima facie assessments of the relationship between two variables, even though correlation as such does not imply a causal relationship. Correlation between each of the six indices and each of the epidemiological variables has been evaluated using Pearson’s linear correlation coefficient. Results are shown in Table 2.

**Table 2:** Contemporaneous bivariate correlation results between each of the indices and the weekly epidemiological variables, for all countries.

Correlation coefficient				
Index	*newCases(t)*	*deaths(t)*	*admICU(t)*	*admHosp(t)*
*BR(t)*	0.220	0.152	0.180	0.017
*HM(t)*	0.132	0.117	− 0.093	− 0.047
*HR(t)*	0.151	0.147	0.268	0.183
*MP(t)*	0.103	0.036	0.020	0.251
*SR(t)*	0.095	0.054	0.092	− 0.052
*SD(t)*	0.241	0.178	− 0.061	0.143

All results are significant ($$p < 0.05)$$. Correlation has been computed pooling all the available countries in a unique vector, for each variable. Business Restrictions (BR), Health Monitoring (HM), Health Resources (HR), Mask Policies (MP), School Restrictions (SR), Social Distancing (SD).

Cross-correlation measures the similarity between a vector *x* and shifted (lagged) copies of a vector *y* as a function of the lag. We calculated cross-correlation for all countries, for each of the six indices with each of the epidemiological variables available for each country. We have conducted these analyses using a lag from − 5 weeks to + 5 weeks. Results are shown in the “Results” section.

#### Granger causality

To further analyze the possible causal effect of the policies summarized by the indices on the epidemiological outcomes (and vice versa), we have subsequently subjected the data to Granger causality tests.

The Granger causality test is a statistical hypothesis test used for determining whether one time series is useful for forecasting another one^20^. When time series *X* Granger-causes time series *Y*, the patterns in *X* are approximately repeated in *Y* after some time lag. Thus, past values of *X* can be used to predict future values of *Y*. The test was performed using the *R* package *lmtest*. The test is a Wald Test; the null hypothesis (H0) is that time series *X* does not Granger-cause time series *Y*, while the Alternative Hypothesis (H1) is that time series *X* Granger-causes time series *Y*. This test generates an F-statistic along with a p-value.

We conducted a Granger causality test for our two main variables of interest, PII and COVID-19 epidemiological outcomes in bivariate analyses (currently, the methods for the generic function *grangertest* only perform a test for Granger causality for bivariate time series). The test has been applied both ways (i.e., estimating the effect of epidemiological variables on the Policy Intensity Indices and vice versa). We used a one-week lag in order to take into account the incubation period of the virus and the weekly aggregation of the data. The test has been carried out in pooled analyses for all nine European countries. Results are shown in “Results” section.

#### Population modelling approach

Building on the work described in the previous sections, we further deepened our investigation of the relationship by implementing autoregressive predictive models on policy and epidemiological data. In particular, we implemented a multilevel vector autoregressive model in order to estimate a single integrated model for all the countries (Eq. 1, where *I* stands for one of the Policy Intensity Indices, and *t* refers to the time indexes defined with a weekly temporal granularity). We considered all countries as an homogeneous group following the paradigm of population modelling, i.e a mathematical/statistical modelling technique that focuses on a population of related individuals, in which each individual is represented by a specific instance of model parameters. The difference in parameter values are due to the inter-individual (or between-subject) variability^21^. In population modelling, we are interested in modelling and describing the typical behavior (central or population tendency) of the model and the variability across the subjects.

1 $$\begin{aligned} {\left\{ \begin{array}{ll} {\textbf {I(t)}} = \beta _{01} + \beta _{11}*I(t-1) + \beta _{21}*newCases(t-1) + \\ \beta _{31}*admH(t-1) + \beta _{41}*admICU(t-1) + \beta _{51}*deaths(t-1) \\ {\textbf {nC(t)}} = \beta _{02} + \beta _{12}*I(t-1) + \beta _{22}*newCases(t-1) + \\ \beta _{32}*admH(t-1) + \beta _{42}*admICU(t-1) + \beta _{52}*deaths(t-1) \\ {\textbf {aH(t)}} = \beta _{03} + \beta _{13}*I(t-1) + \beta _{23}*newCases(t-1) + \\ \beta _{33}*admH(t-1) + \beta _{43}*admICU(t-1) + \beta _{53}*deaths(t-1) \\ {\textbf {aICU(t)}} = \beta _{04} + \beta _{14}*I(t-1) + \beta _{24}*newCases(t-1) + \\ \beta _{34}*admH(t-1) + \beta _{44}*admICU(t-1) + \beta _{54}*deaths(t-1) \\ {\textbf {d(t)}} = \beta _{05} + \beta _{15}*I(t-1) + \beta _{25}*newCases(t-1) + \\ \beta _{35}*admH(t-1) + \beta _{45}*admICU(t-1) + \beta _{55}*deaths(t-1) \\ \end{array}\right. } \end{aligned}$$

In our model, we consider each country as a single individual of a population, where the population is the set of the 9 selected European countries. Each country has its own individual model, which is assumed to be a slight variation from the common population model. In particular, the population parameters (i.e. the same for all the individuals of the population) are named *fixed effects*, whereas *random effects* are defined as parameters conditional to the particular subject and are added to the common population model. The underlying idea is that the parameters of these models are extracted from the same distribution, assumed to be Gaussian with mean equal to the estimated population mean and standard deviation equal to the one calculated from the contributes of the countries (Eq. 2).

2 $$\begin{aligned} \beta _{ij} \sim N(\beta _{j,pop},\sigma _{\beta _{j,pop}}) \end{aligned}$$

Equation (1), also fits the definition of a multivariate autoregressive model, where all the variables at time *t* only depend from the ones at the previous temporal interval $t-1$. The multilevel vector autoregression has been implemented using *RStudio*, with the dedicated *R* packages *mlVAR*^22^ and *lme4*^23^.

To make sure that one week was the optimal lag for our model, we tested it using both the Akaike Information Criteria (AIC) and Bayesian Information Criterion (BIC) on the mlVAR model with lag = 1, 2 and 3. For both criterions, lag = 1 minimizes the measures, hence is the best lag value. The values are reported in Supplemental material 2.

The input to the model parameter estimation phase consists of a data table containing one variable from the PIIs (we built six different datasets of the population for each of the six different PII), and the four chosen epidemiological parameters (*new cases, hospital admissions, ICU admissions, deaths*), with an additional column specifying the ID of the country that can be used to separate the random effects.

The output is the estimate of the regression parameters of the autoregressive model.

The following equation represents an example for the Business Restriction PII area (Eq. 3):

3 $$\begin{aligned} {\begin{matrix} I_{BR}(t) \sim Predictor1 + Predictor2 + Predictor3 + Predictor4 + \\ Predictor5 + Predictor7 + Predictor8 + Predictor9 + \\ Predictor10 + ((1 | ID) + (Predictor1 | ID) + \\ (Predictor2 | ID) + (Predictor3 | ID) + \\ (Predictor4 | ID) + (Predictor5 | ID)) \end{matrix}} \end{aligned}$$

where the predictors from 1 to 5 are related to the variables at $t-1$, i.e. to the temporal effects, while predictors from 7 to 10 are related to the contemporaneous effects, i.e. the simultaneous effects of other variables at time *t*. These are shown in Eq. (4) and in Table 3. Note that the conditional part of the formula refers to the Random Effects: their contribution is conditional to the ID of the country.

4 $$\begin{aligned} {\begin{matrix} I_{BR}(t) \sim BR(t-1) + newCases(t-1) + admHosp(t-1) + \\ admICU(t-1) + deaths(t-1) + newCases(t) + admHosp(t) + \\ admICU(t) + deaths(t) + ((1 | ID) + (BR(t-1) | ID) + \\ (newCases(t-1) | ID) + (admHosp(t-1) | ID) + \\ (admICU(t-1) | ID) + (deaths(t-1) | ID)) \end{matrix}} \end{aligned}$$

Since contemporaneous effects are estimated post-hoc from the residuals by the algorithm, we decided to focus only on the temporal and random effects.

**Table 3:** *mlVAR* dependent and predicted variables, the lags considered for each, and the predictor IDs used in the analysis.

Dependent variable	Predicted variable	Temporal lag	Predictor ID
$I_{score}$	$I_{score}$	1	Predictor1
*newCases*	$I_{score}$	1	Predictor1
*admHosp*	$I_{score}$	1	Predictor1
*admICU*	$I_{score}$	1	Predictor1
*deaths*	$I_{score}$	1	Predictor1
$I_{score}$	*newCases*	1	Predictor2
*newCases*	*newCases*	1	Predictor2
*admHosp*	*newCases*	1	Predictor2
*admICU*	*newCases*	1	Predictor2
*deaths*	*newCases*	1	Predictor2
$I_{score}$	*admHosp*	1	Predictor3
*newCases*	*admHosp*	1	Predictor3
*admHosp*	*admHosp*	1	Predictor3
*admICU*	*admHosp*	1	Predictor3
*deaths*	*admHosp*	1	Predictor3
$I_{score}$	*admICU*	1	Predictor4
*newCases*	*admICU*	1	Predictor4
*admHosp*	*admICU*	1	Predictor4
*admICU*	*admICU*	1	Predictor4
*deaths*	*admICU*	1	Predictor4
$I_{score}$	*deaths*	1	Predictor5
*newCases*	*deaths*	1	Predictor5
*admHosp*	*deaths*	1	Predictor5
*admICU*	*deaths*	1	Predictor5
*deaths*	*deaths*	1	Predictor5
*newCases*	$I_{score}$	0	Predictor6
*admHosp*	$I_{score}$	0	Predictor6
*admICU*	$I_{score}$	0	Predictor6
*deaths*	$I_{score}$	0	Predictor6
$I_{score}$	*newCases*	0	Predictor7
*admHosp*	*newCases*	0	Predictor7
*admICU*	*newCases*	0	Predictor7
*deaths*	*newCases*	0	Predictor7
$I_{score}$	*admHosp*	0	Predictor8
*newCases*	*admHosp*	0	Predictor8
*admICU*	*admHosp*	0	Predictor8
*deaths*	*admHosp*	0	Predictor8
$I_{score}$	*admICU*	0	Predictor9
*newCases*	*admICU*	0	Predictor9
*admHosp*	*admICU*	0	Predictor9
*deaths*	*admICU*	0	Predictor9
$I_{score}$	*deaths*	0	Predictor10
*newCases*	*deaths*	0	Predictor10
*admHosp*	*deaths*	0	Predictor10
*admICU*	*deaths*	0	Predictor10

To verify the validity of the convenience of differentiating between fixed and random effects, we performed a Hausman test^24^, the results of which are reported in Table 4. It can be noticed that for all the six indices, the use of random effects is preferred, as the null hypothesis is never rejected.

**Table 4:** p-values for Hausman test performed to decide between fixed or random effects.

PII	BR	HM	HR	MP	SD	SR
*p-value*	0.544	0.983	0.838	0.576	0.999	0.944

The null hypothesis is never rejected, so the mlvar model properly makes use of random effects.

Before running the regression model, we tested for the assumptions of absence of multicollinearity, homoskedasticity and exogeneity. Multicollinearity among the independent variables was assessed calculating the Tolerance (the percent of the variance in the independent variable that cannot be accounted for by the other independent variables) and Variance Inflation Factors (VIF, measures the inflation in the coefficient of the independent variable due to the collinearities among the other independent variables). Multicollinearity does not influence the precision of predictions, nor the goodness-of-fit statistics, but could be a problem when drawing conclusions about independent variables. The results of our test are in Table 5. A VIF of 1 means that the regression coefficient is not inflated by the presence of the other predictors, hence multicollinearity does not exist. As a rule of thumb, a VIF exceeding 5 requires further investigation, whereas VIFs above 10 indicate multicollinearity. Ideally, the Variance Inflation Factors are below 3.

**Table 5:** The Tolerance is the percent of the variance in the independent variable that cannot be accounted for by the other independent variables.

Variables	Tolerance	VIF
new Cases	0.458	2.184
adm Hosp	0.117	8.552
adm ICU	0.112	8.927
deaths	0.569	1.758

The Variance Inflation Factor (VIF) measures the inflation in the coefficient of the independent variable due to the collinearities among the other independent variables.

Results are borderline for the variables related to hospitalization rates, as the tolerance values are close to the 0.1 threshold and the VIFs indicate that there might be a moderate correlation, although they do not suggest evident multicollinearity. This could mean that the two variables are more correlated in some countries than in others, as suggested also by the correlations calculated in the “Missing data imputation” section. Since we are implementing a mixed-effects model that takes into account both the population factors and the individual differences, we decided to keep all the variables in order to have an estimation of the contributions of all the countries, including those for which the two variables *adm Hosp* and *adm ICU* are not correlated.

Homoskedasticity has been tested with the Breusch-Pagan test, and results show that the assumption of homoskedasticity cannot be confirmed. However, differently from standard linear regressions, heteroskedasticity in mixed-effects models is not a big issue, as the presence of random effects already takes into account the different variances of the groups.

Regarding exogeneity, it should be present by design as our independent variables at time $(t-1)$ cannot be dependent on the dependent variables at time (*t*).

Plus, linear mixed-effects models have proven to be robust to one or more violations to the assumptions made in standard linear regression^25,26^.

### Ethical approval

Ethical approval in this work has not been sought since we worked on data publicly available.

## Results

### Imputed missing data

**Figure 2:**
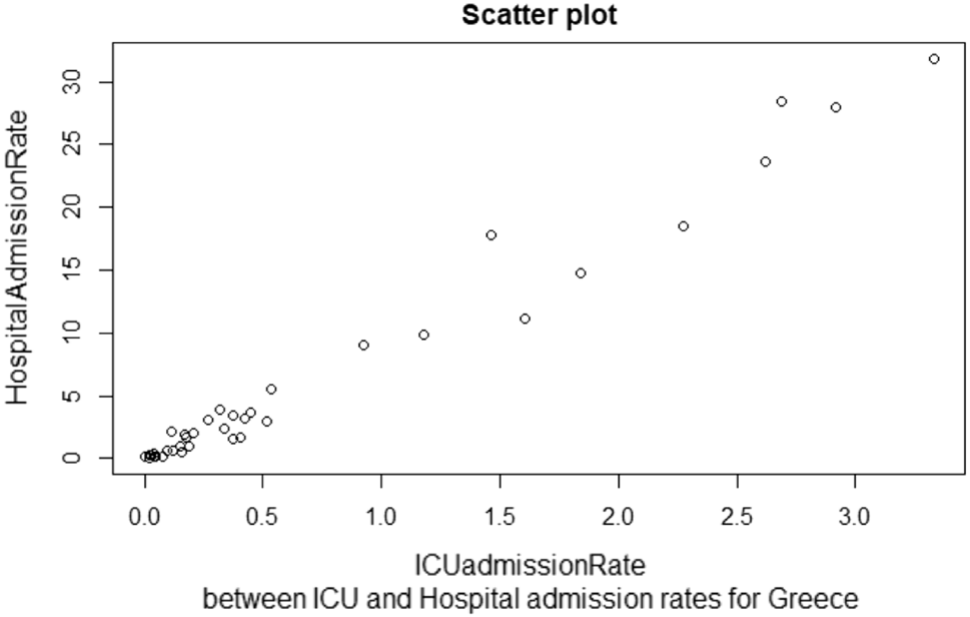
Example of correlation between the variables *admICU* and *admHosp* from Greece.

As seen in “Methods” section, four additional countries needed imputation: Greece, Ireland, Cyprus and Estonia, with at most 30% of missing values for only one variable. We proceeded by imputing the missing values with a univariate or bivariate regression model. The imputing model has been chosen to be suitable to predict missing values in longitudinal data, and it is justified by the correlation analysis between the missing data variables and the other attributes presenting complete data. For example, considering data for Greece, which were missing 7 values from the *admHosp* variable, it was observed that the correlation with the variable *admICU* was high, being 0.987 (scatter plot in Fig. 2), so *admICU* has been chosen as a regressor for Greece’s univariate regression model.

This correlation analysis has been done for each of the selected countries. Predictors for the regression have been selected if the missing data variables had a correlation greater than 0.6 with the other variables of the dataset, leading to imputing values as in Table 6, where the models used for imputing missing values for the selected countries (Greece, Ireland, Cyprus and Estonia) are shown, together with the correlation with the variables used as regressors of the imputation model.

**Table 6:** Imputation models for the missing values of the variable “Missing variable” for the four shown countries, and the correlation value with the predictor variables.

Country	Missing variable	Correlation value	Chosen model
Greece	*admHosp*	0.99, *admICU*	Univariate
Ireland	*admICU*	0.63, *admHosp* 0.80, *deaths*	Bivariate
Cyprus	*admICU*	0.85, *admHosp* 0.88, *deaths*	Bivariate
Estonia	*admICU*	0.71, *admHosp*	Univariate

### Correlation and cross-correlation analysis

We show the results for the raw correlation analysis between the PIIs and different epidemiological variables without any time lags in Table 2. Except for a few values, correlation results are mostly positive but with low magnitude, if we consider that from 0.0 to 0.29 is negligible correlation, from 0.3 to 0.49 is low correlation, from 0.50 to 0.69 is medium correlation, and from 0.70 to 1.00 is considered high correlation^27^. Indeed, only a few correlation values exceed 0.2, e.g. between *Health Resources* and *Weekly ICU admission rates* (0.268), or *Mask Policies* and *Weekly Hospital admission rates* (0.251).

These low correlations were expected, as without time lags, the raw correlations do not account for the COVID-19 incubation period, i.e. the number of days between the contact with the virus and the onset of symptoms. According to available research, on average, in the first variant of the disease symptoms show up in a newly infected person about 5.6 days after contact^28^. Most people with symptoms had them by day 12, and almost all have them by day 14, though in rare cases, symptoms appeared as soon as 2 days after exposure. The lack of time lags also means that the analyses cannot account for the delay in reporting the number of the cases; this delay would contribute to produce a further temporal lag between the application of a new policy and its effects on the epidemiological variables. On top of that, there is usually also a delay between testing positive and eventually being hospitalized, so the effects are visible later and this contributes to increase the temporal lags^29^.

**Figure 3:**
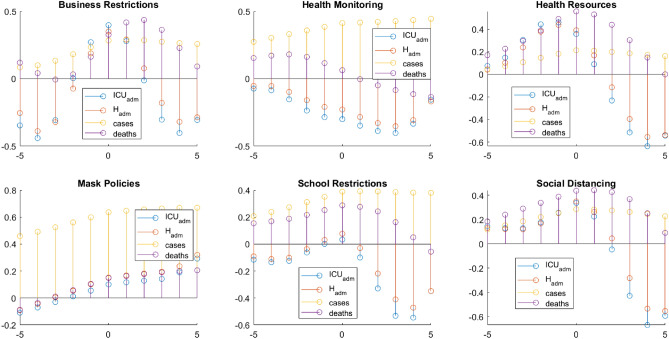
Cross-correlation between indices and epidemiological variables for France. *x* lag value, *y* cross-correlation value.

These findings motivated us to pursue a slightly different correlation analysis, which considers a temporal lag and computes the *cross-correlation*. Figure 3 shows an example of this type of analysis for France with its estimated cross-correlation for each of the six Policy Intensity Indices with the available epidemiological variables. Figures with additional results related to other countries can be found in Supplemental Material 1. The *x*-axis represents the temporal lag (in weeks): lag zero means that no temporal lag is assumed; a positive lag reflects the effect of the indices on the variables, while a negative lag reflects the effect of the variables on the policies, i.e. on the PIIs. The largest spike in the *y*-axis occurs where the lag is such that elements of *x* and *y* match the most. The purpose of this analysis was to see if each of the six PIIs (BR, HM, HR, MP, SR, SD) has an effect on the epidemiological variables (*Hospital admission rate*, *ICU admission rate*, *new cases* and *deaths*) in the weeks following the enactment of new policies. It is thus possible to visually inspect the trend of the spikes in the positive temporal lags, from 1 to 5. At the same time, since we are also interested in seeing how the evolution of the epidemiological variables influenced the policymakers, this can also be accomplished looking at the trends of the spikes in the negative temporal lags from $-1$ to $-5$ (the left side of the plots).

With regards to France in particular, that shows the most clearly interpretable results (Fig. 3)—but we can observe the same phenomena for all the analyzed countries—we note that the highest spikes do not always correspond to lag 0. This confirms the lagged effect of the PIIs on the epidemiological variables and vice versa, so PIIs do have a high correlation with the epidemiological variables, but shifted in time. Second, for the *Health Resources* index, there is a trend that reverses the value of the spikes from negative lag to positive lag, as we can see the values shifting gradually from positive correlation to negative correlation, for the variables *ICU admission rate* and *Hospital admission rate*. This trend suggests that the effort put in providing more medical equipment showed somehow an improvement in the reduction of hospitals and intensive care units occupancy. Same conclusions for *Social Distancing*, *Business Restrictions* and *School Restrictions*. Looking at negative lags for HR, it also seems that an increase in *cases*, *deaths* and *hospital* and *ICU admissions* led mostly to take extra measures (positive and increasing correlation), and we can see the indices as an effect rather than a cause. Going toward lag zero, they start to decrease rapidly until a subsequent shift in the sign, indicating that the measure of HR is actually improving the situation.

Another interesting finding is that *Health Monitoring* and *new cases* are positively cross-correlated, and the values of correlation grow in time; these are the consequence of an augmented monitoring of individual health statuses, like contact tracing, temperature checks, test or questionnaires. These results suggest that having consciousness of one own health status is more likely to lead to immediate care, which can avert the worsening of health conditions and prevent increased numbers of *deaths*.

### Granger causality interpretation

The results of the Granger Causality analysis, focusing on the significant results ($p<0.05$), are synthesized in Tables 7 and 8. Table 7 shows that the variables *hospital admission rate* and *ICU admission rate* have an important Granger-causal influence on the enactment of policies, since at least one country has a significant p-value for each index. The findings in Table 8, indicated that the outcomes that appear to have been most influenced by the policies, as summarized by the indices, are *ICU admission rate*, followed by *hospital admission* and *deaths rate*. Maybe most importantly, the analyses do not allow us to conclude that *X* is primarily causing *Y* rather than *Y* causing *X*. There appears to be a more complex two-way relationship at work, with PIIs influencing the epidemiological outcome variables and vice versa, for example in Table 8 the highest influence of all the PIIs is in the variable *ICUadm*, with a total of 13, but also in Table 7 the same variables is the most influencing in all the PIIs, with a total of 12. So we could say that, e.g., hospitalizations and deaths influenced the PIIs (maybe because the governments started to act when they observed severe changes in the health services sector) but then the policies started to influence the trend of epidemiological outcomes as well.

**Table 7:** Number of countries (out of the 9 taken into consideration) that have a significant p-value ($<0.05$) on their Granger causality, where each of the four epidemiological variables Granger-causes each of the six indices, with a lag of 1 week.

Granger causality					
	New Cases	adm Hosp	adm ICU	Deaths	Tot.
**BR**	0	2	3	3	8
**HM**	3	3	1	2	9
**HR**	0	1	2	0	3
**MP**	3	3	1	0	7
**SR**	0	1	2	3	6
**SD**	0	2	3	1	6
Tot.	6	12	12	9

**Table 8:** Number of countries (out of the 9 taken into consideration) that have a significant p-value ($<0.05$) on their Granger causality, where each of the six the indices Granger-causes each of the four epidemiological variables, with a lag of 1 week.

Granger causality							
	BR	HM	HR	MP	SR	SD	Tot.
**New** **Cases**	0	0	1	1	0	0	2
**adm Hosp**	1	2	4	2	0	1	10
**adm ICU**	1	3	4	2	2	1	13
**Deaths**	3	0	1	2	1	2	9
Tot.	5	5	10	7	3	4	

### Population modelling fixed and random effects

Results for the fixed effects population are shown in Table 9 for the Business Restriction index which is taken as an example; other results are reported in the Supplemental material 2. The table shows the estimated value of the parameters of the model that defines $BR(t) \sim$, with their associated standard error (SE) and p-value.

From this table, we can observe how the strongest predictor of Business Restrictions, are the Business Restrictions from the previous week $I(t-1)$ (and has a *p-value*. As is shown in the Supplemental material 2, this behaviour can be observed for the other PIIs as well. Aside from this, we can see that there appears to be a positive significant relationship between $admICU(t-1)$ and business restrictions, while there is a negative and significant relationship between $deaths(t-1)$ and business restrictions. These results suggest that while an increase in the number of ill people appear to be associated with more investment in business restrictions, an increase in the number of deaths from COVID-19 appears to be associated with less policy investment in business restrictions. These results could be related to the fact that most of the deaths are of the elderly population (most vulnerable to COVID-19) which is not active in the labor force, and our suggestion is that it may be of interest to estimate models which can distinguish deaths and ICU admissions by age. From our autoregressive model, we are able to quantify the distribution of the population parameters, assumed to be drawn from a Normal distribution. For the index Business Restrictions, we obtain in Eq. (5) the following distributions for the prediction of *I*(*t*) (Eq. 4):

5 $$\begin{aligned} {\begin{matrix} \beta _{01} \sim N(0.033,0.53558) \\ \beta _{11} \sim N(0.712,0.21979) \\ \beta _{21} \sim N(-0.036,0) \\ \beta _{31} \sim N(-0.011,0) \\ \beta _{41} \sim N(0.115,0.06495) \\ \beta _{51} \sim N(-0.121,0.08780) \\ \end{matrix}} \end{aligned}$$

So we have the intercept $\beta _{01}$ which is close to 0 but has a higher variance with regards to the other parameters, $\beta _{11}$ has the highest mean and in fact it refers to the previous value in time of the PII. $\beta _{21}$ and $\beta _{31}$ have a low negative mean but a variance which is practically 0, so *newCases* and *admHosp* behave the same for all the 9 countries in modelling the BR PII. Regarding the relationships between other PIIs (see Supplementary material 2), in terms of the most notable findings, We observe that there is a significant negative relationship between $deaths(t-1)$ and Social Distancing. Mask Policies has a significant negative correlation with $admICU(t-1)$. School Restrictions and Health Monitoring do not show any coefficient with a significant *p-value*.

Furthermore, we show the parameter estimates for the models run with random effects in Table 10. In these models, each country is given the contribute that has to be added to the population parameter. From the magnitude of these contributions, we can distinguish which countries deviate more from the population model. For example, we can see that for the Business Restriction models, Latvia and Ireland deviate more with a positive random effect, while Greece and the Netherlands deviates more with a negative random effect, both for the *Intercept* value, while all the other parameters are quite stable between countries.

Tables showing results for all the other PIIs can be found in Supplemental material 2. In general, regarding the random effects parameter estimates for the epidemiological outcome variables, we find variations in terms of how much countries deviate from the common population model. For instance, no country significantly contributes more than others to the common population model with regards to $newCases(t-1)$ and $admHosp(t-1)$ for the Business Restrictions, School Restrictions and Social Distancing PIIs, and a large inter-country variability can be observed for all variables at $t-1$ for the Health Monitoring and Health Resources PIIs. The results of the contribution of random effects suggest that there was no common agreement on the level of commitment in the different policy areas among countries, as each country responded individually to the epidemiological situation.

**Table 9:** Fixed effects of the population model for the Business Restriction considered as Policy Intensity Index.

Fixed effects $BR(t) \sim$			
Parameter	Value	SE	p-value
*Intercept*	0.033	0.536	0.855
$I(t-1)$	0.712	0.083	0.000
$newCases(t-1)$	− 0.036	0.019	0.060
$admHosp(t-1)$	− 0.011	0.051	0.836
$admICU(t-1)$	0.115	0.051	0.024
$deaths(t-1)$	− 0.121	0.042	0.004

**Table 10:** Random effects of the population model for the Business Restriction considered as Policy Intensity Index.

Random effects $BR(t) \sim$						
ID	Intercept	$I_{t-1}$	$newCases_{t-1}$	$admHosp_{t-1}$	$admICU_{t-1}$	$deaths_{t-1}$
Spain	0.003	0.196	0	0	0.065	0.066
France	− 0.052	− 0.096	0	0	0.0382	− 0.108
Netherlands	− 0.468	− 0.054	0	0	0.080	− 0.072
Latvia	0.773	− 0.032	0	0	− 0.025	0.007
Slovenia	0.190	− 0.469	0	0	− 0.057	0.109
Greece	− 1.092	0.140	0	0	− 0.0003	0.031
Ireland	0.619	0.182	0	0	− 0.048	− 0.064
Cyprus	− 0.251	0.002	0	0	0.002	0.009
Estonia	0.280	0.133	0	0	− 0.055	0.021

## Discussion

Our analysis process started with the investigation of simple correlations, which proved useful both for the sake of a preliminary understanding of the interactions among PIIs and epidemiological variables, as well as to devise the best imputation strategy for missing data, which allowed us to have complete data for 9 countries, and include them in the further analysis steps.

Correlation analysis identified low correlation between PIIs and epidemiological variables. This is indicative of the fact that an action on PIIs at time *t* is not correlated to the value of an epidemiological variable at the same time *t* (and vice versa), confirmed in concordance to findings in article^29^.

As a second step in the analysis pipeline, we investigated the relationship between variables, using cross-correlation, that is able to account for a time difference (between − 5 and + 5 weeks, in our specific case) or “lag” between a perturbation in one time-series and its effect on a dependent one. The cross-correlation analyses revealed medium to high correlations between the epidemiological outcome variables and the PIIs. Cross-correlation is a more effective tool to investigate such relations since a temporal delay between cause and effect is a reasonable hypothesis considering factors such as the incubation period of the virus, the delay in reporting case counts or PCR test results.

The main findings of the cross correlation analysis consist in the fact that a lag is needed to find medium to high correlation values between PIIs and epidemiological variables time series. The most common results are high negative correlation ($<-0.5$) for Health Resources PII on the *Hospital admission* variable, after 2 weeks of time lag, as well as Business Restrictions. A special case is reserved to the *Cases* variables, which show a positive correlation to Health Resources. This is explained by the fact that a bigger effort in this field allows a bigger detection of COVID-19 cases. The most similar cross-correlation paths are found for Business Restrictions, Health Resources (beside *Cases* as mentioned before) and Social Distancing, especially toward the *Hospital* and *ICU admission* variables.

As a final step, motivated by the results of the previous phases in our analyses, we employed an autoregressive model to find the relation between time series values depending on the previous values of the series itself, using Multilevel Autoregression. Such population modelling approach has the advantage of eliciting common patterns that are shared by all the 9 countries analyzed, while also accounting for country-specific phenomena explained by the random effects component. While being potentially less accurate for any single country compared with a stand-alone model for each country, the population model is able to highlight the most important factors on the overall pooled population. In our analyses, such factors were the number of new cases and deaths that mostly influenced the PIIs, as shown in Supplementary material 2. There, we can also see that results from this method show that Health Monitoring and Health Resources indices had the greatest impact on containing COVID-19, confirming that putting more effort in countries’ Health Systems actually helped control the pandemic. Business Restrictions appear to have had a significant effect, too, probably because these restrictions very directly reduced opportunities for the virus to spread from infected to not-yet-infected people by severely limiting possibilities for people to meet or gather, and notice that all the 9 countries for Business Restrictions responded in a uniform way to the incidence of *New Cases* and to the incidence of *Hospital Admissions*, as there are no random contributes but they share the same parameter value as the common population model, as seen in Table 10.

Two limitations should be noted: First, our analysis sought to build predictive models for a comprehensive set of European countries; unfortunately due to missing data for several weeks or even entirely missing variables for specific countries, we had to limit our analysis to 9 different European countries.

Second, as mentioned when we introduced the Policy Intensity Indices, concerns the meaning of those indices. It is important to stress that a high score corresponds to a major effort in managing specific policies: the more policies are announced or updated, the higher the PII assigned. However, a high PII does not necessarily mean that the country had particularly strict policies. Indeed, an update to a Mask Policy, allowing citizens to be mask-free outdoor (i.e., relaxing a previously imposed requirement of wearing a mask outdoor) would still increase the Mask Policies PII. This implies that countries with the same PII value could actually have a different situation policy-wise, and that would increase the intra-individual variance considerably.

It is important to acknowledge that a policymaker ultimately needs to consider larger trade-offs: a policy that is highly effective against the spread of COVID-19 might nonetheless be so economically, politically, or otherwise costly that it—appropriately—will not be chosen. In this paper we do not analyze secondary/unintended non-epidemiological effects.

## Conclusion

The main purpose of this study was to provide an approach to analyze how helpful six broad categories of non-pharmaceutical interventions (NPIs), adopted by many governments to varying degrees, have been to contain the spread of the virus across Europe during the first year.

To keep track of the efforts by governments in enacting new policies and decisions across the six kinds on NPIs, we used the Policy Intensity Indices on Business Restrictions, Health Monitoring, Health Resources, Mask Policies, School Restrictions and Social Distancing, which summarize how policymakers dealt with the evolving pandemic. The epidemiological variable of interest have been *New cases* and *deaths*, *Hospital admission rate* and *ICU admission rate*. Results show that the inter-country variability of the effects of all indexes is generally high, but generally speaking, health monitoring and health resources policies seem to have been the most effective in containing the effects of the pandemic on a European scale.

A future direction for follow-up work could consist in collecting data for more countries and consider also 2021 data, which may now be available. However, including 2021 would require also tackling new factors, like the vaccination campaign, more recent viral variants, and the interactions of these two factors. This may require the introduction of more complex models, and a need to carefully evaluate the effects of pooling data from a sample affected by significant population shift over time.

## Supplementary Information

Supplementary Information 1.

Supplementary Information 2.

## Data Availability

The PERISCOPE Atlas is constructed over a database composed of data integrated from different external sources, including both public data and data from regional authorities that support the project. Health data are acquired by the European Centre of Disease Control and Prevention (ECDC, the source of the health data used in our analyses) and Johns Hopkins University. The link to the online atlas is: *atlas.periscopeproject.eu*.
