# Supplementary material for: A comparative analysis of the effects of containment policies on the epidemiological manifestation of the COVID-19 pandemic across nine European countries
Source: Sci Rep. 2023 Jul 19;13:11631. doi: 10.1038/s41598-023-37751-4 (PMC10356910; doi:10.1038/s41598-023-37751-4)
Supplement: Supplementary file 1 — Supplementary Information 1. [file 41598_2023_37751_MOESM1_ESM.pdf]

Supplementary 1

1 Cross-Correlation Results

This section reports further results for cross-correlation analyses.
Figure 1 for France, Figure 2 for Germany, Figure 3 for Italy, Figure 4 for Spain, Figure 5 for United Kingdom. We show the results for some of the biggest European countries, even though Germany and Italy are missing information for the variable *admICU*, and United Kingdom is missing information for both variables *admHospital* and *admICU*.

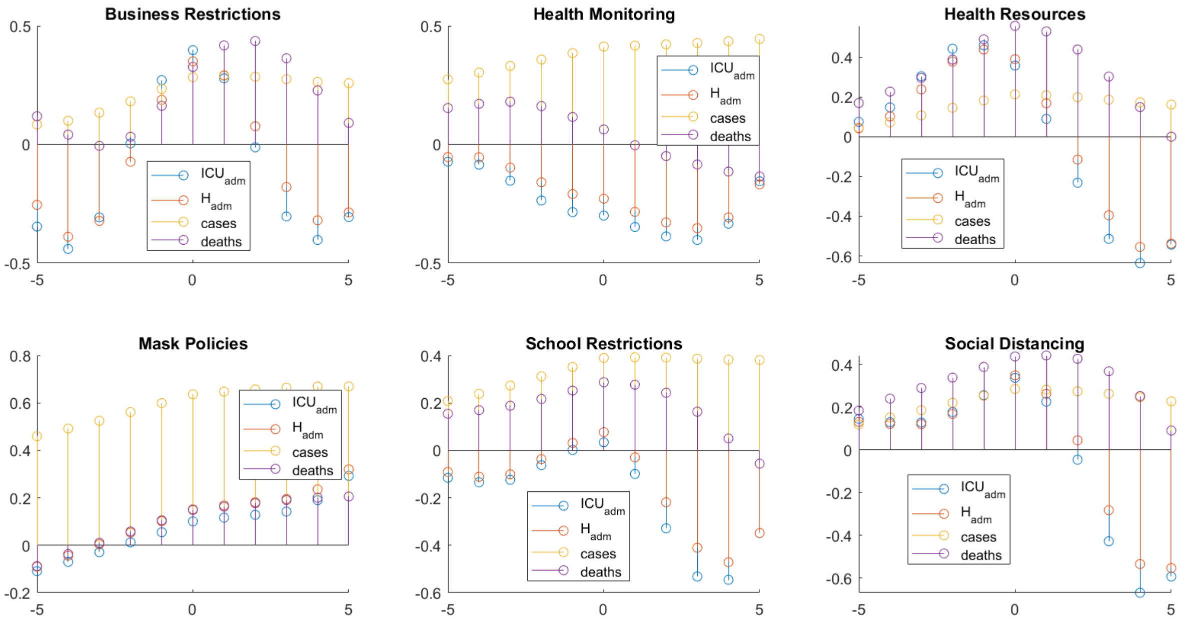

Figure 1. Cross-correlation between indices and epidemiological variables for France. *x* lag value, *y* cross-correlation value

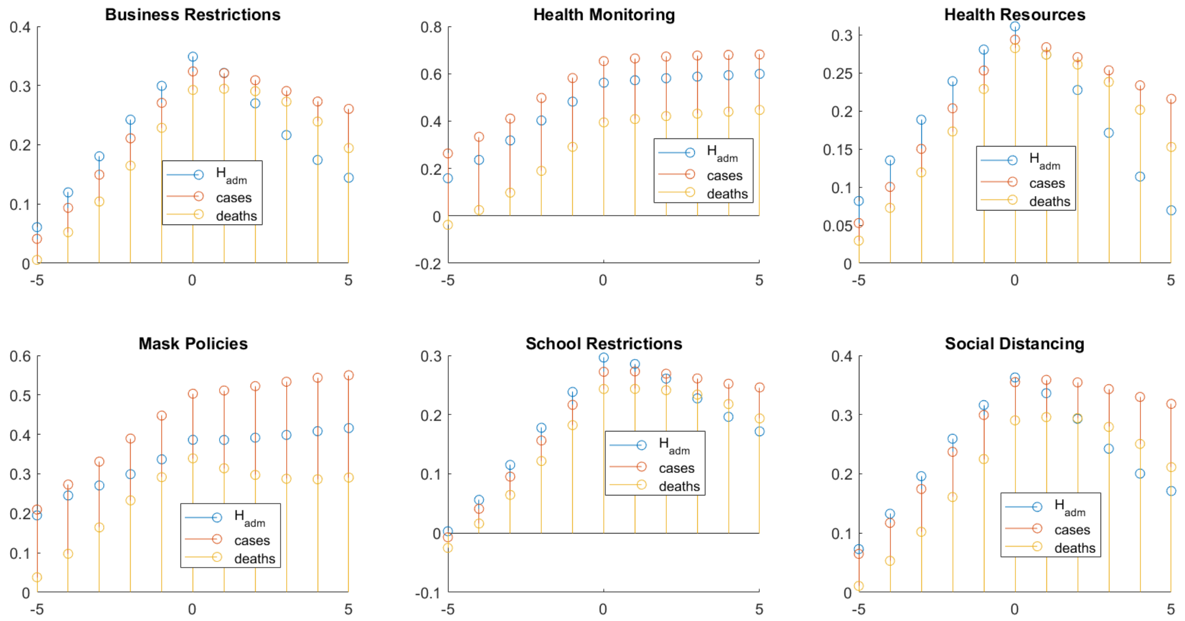

Figure 2. Cross-correlation between indices and epidemiological variables for Germany. *x* lag value, *y* cross-correlation value

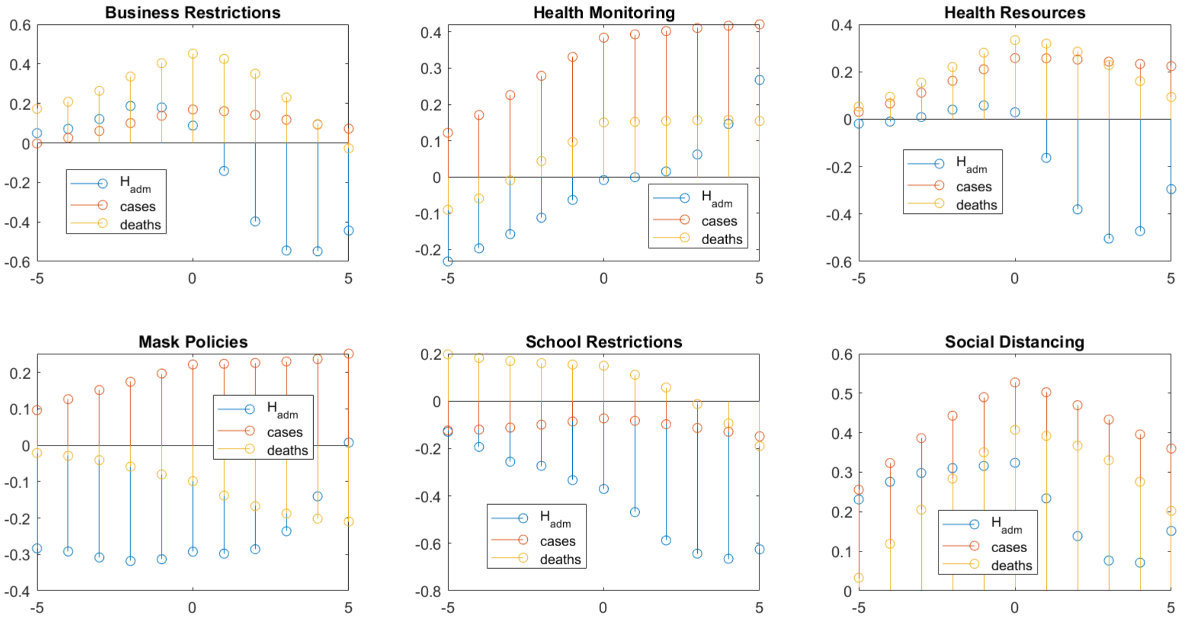

Figure 3. Cross-correlation between indices and epidemiological variables for Italy. *x* lag value, *y* cross-correlation value

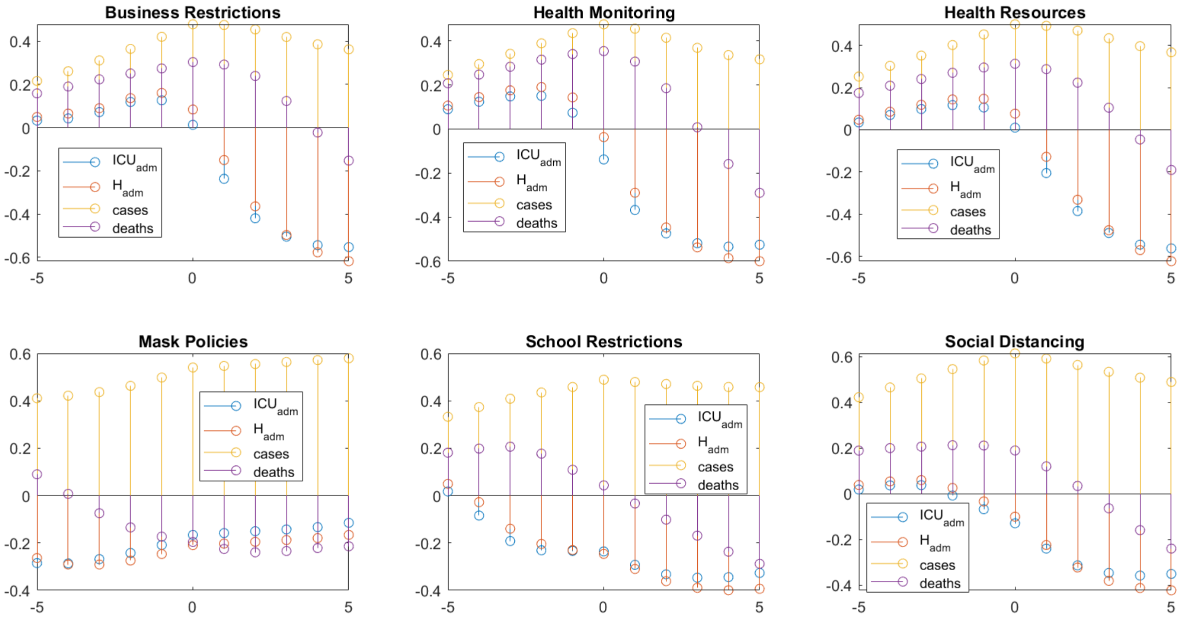

Figure 4. Cross-correlation between indices and epidemiological variables for Spain. *x* lag value, *y* cross-correlation value

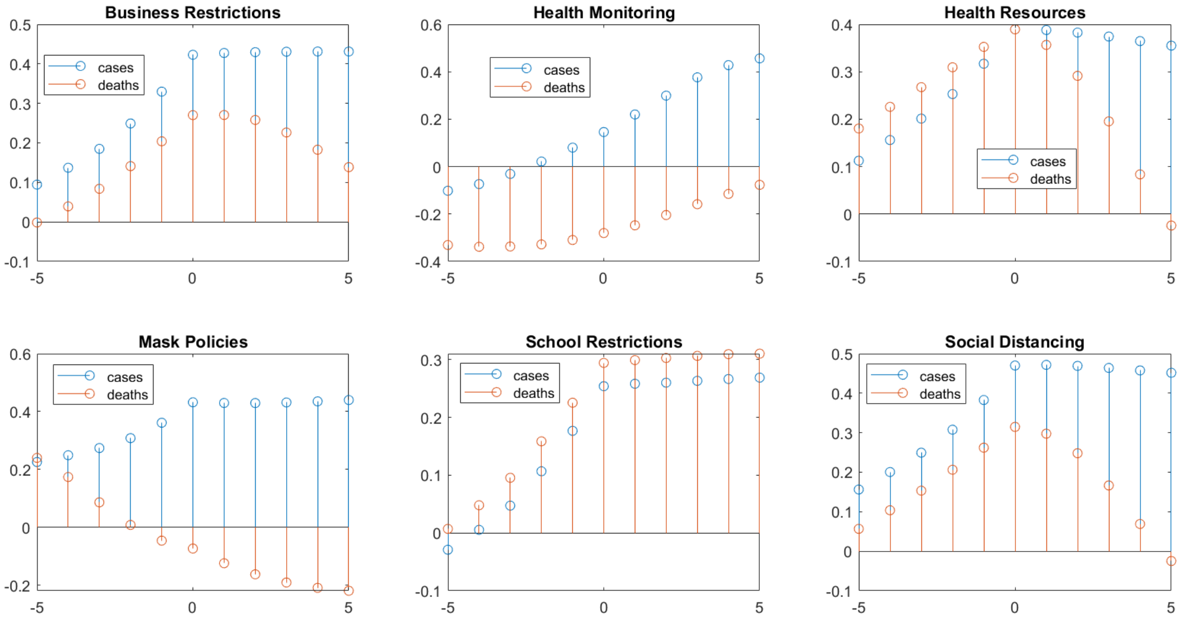

Figure 5. Cross-correlation between indices and epidemiological variables for UK. *x* lag value, *y* cross-correlation value
