# Supplementary material for: A comparative analysis of the effects of containment policies on the epidemiological manifestation of the COVID-19 pandemic across nine European countries
Source: Sci Rep. 2023 Jul 19;13:11631. doi: 10.1038/s41598-023-37751-4 (PMC10356910; doi:10.1038/s41598-023-37751-4)
Supplement: Supplementary file 2 — Supplementary Information 2. [file 41598_2023_37751_MOESM2_ESM.pdf]

Supplementary 2

1 Fixed and Random Effects

In this section I report more results for the Fixed and Random Effects of the population model for the remaining predictive models for the indices: Health Monitoring (Tables 2 and 3), Health Resources (Tables 4 and 5), Mask Policies (Tables 6 and 7), School Restrictions (Tables 8 and 9), Social Distancing (Tables 10 and 11), and again, Business Restrictions (Tables 12 and 13). Table 1 shows the AIC and BIC results from the comparison between lag=1,2,3 for the mlvar models.

It is interesting to note that for all the models, in the **Fixed Effects**, the intercept has a very high *p-value*, so the constant effect that is found is not significant.
Instead, the lowest *p-value* is always associated to the Policy Intensity Index at time $t-1$, and that is consistent with the fact that we do not expect the policy to change drastically from one week to another, so indeed the value of $I(t)$ will be for sure influenced by the value of $I(t-1)$, and the value of the coefficients for all the models are positive and higher than the other coefficients.
Other significant coefficients, thus with a *p-value* < 0.05, are the one for $newCases(t-1)$ ($p = 0.044$) for Health Resources, meaning that a strong indicator for making decision in that context is the simple trend of the infections; $admHosp(t-1)$ ($p = 0.035$) and $deaths(t-1)$ ($p = 0.04$) have a significant coefficient for Social Distancing. In Business Restrictions, there are low *p-values* associated to $admICU(t-1)$ ($p = 0.024$) and $deaths(t-1)$, ($p = 0.004$).
Health Monitoring has the coefficients for $deaths(t-1)$ with $p = 0.118$, and Mask Policies $newCases(t-1)$ with $p = 0.076$, while School Restrictions does not show any coefficient with a significant *p-value*.
In all the cases, the coefficients have values close to zero.
About the **Random Effects** results, there is an interesting feature on which to focus: for some variables, there is no random effects variability between the individuals. For example, in Mask Policies, the variable $deaths(t-1)$ is considered with the same influence for all the countries, as well as $newCases(t-1)$, $admHosp(t-1)$, $admICU(t-1)$ for School Restrictions and Social Distancing; $newCases(t-1)$ and $admHosp(t-1)$ for Business Restrictions. The indices Health Monitoring and Health Resources show inter-individual variability for all the variables at $t-1$.

I(t) mlvar model	**Business Restrictions**		**Health Monitoring**		**Health Resources**		**Mask Policies**		**School Restrictions**		**Social Distancing**	
	AIC	BIC	AIC	BIC	AIC	BIC	AIC	BIC	AIC	BIC	AIC	BIC
lag 1	**-44.9**	**21.5**	**-938.2**	**-871.7**	**-707.7**	**-641.1**	**-204.7**	**-138.1**	**-9.3**	**57.1**	**-282.7**	**-216.2**
lag 2	162.5	228.6	-587.9	-521.7	-269.9	-203.8	157.5	223.6	151.0	217.1	28.1	94.2
lag 3	179.5	245.2	-315.9	-250.2	-118.2	-52.5	262.2	327.9	111.2	176.9	162.0	227.7

Table 1. Lag comparison for *I(t)* mlvar models between lag = 1, 2, 3. Lowest AIC and BIC detect the best model between the three performed.

Fixed Effects $HM(t) \sim$

**Parameter**	**value**	**SE**	**p-value**
$Intercept$	0.015	0.519	0.932
$I(t-1)$	0.688	0.358	3.92e-04
$newCases(t-1)$	-0.017	5.659e-02	0.458
$admHosp(t-1)$	-0.0502	0.129	0.335
$admICU(t-1)$	0.082	0.246	0.332
$deaths(t-1)$	0.024	0.032	0.118

Table 2. Fixed effects of the population model for the **Health Monitoring** considered as Policy Intensity Index

Random Effects $HM(t) \sim$

**ID**	**Intercept**	I_{t-1}	$newCases_{t-1}$	$admHosp_{t-1}$	$admICU_{t-1}$	$deaths_{t-1}$
Spain	0.254	0.323	-0.134	-0.312	0.677	0.045
France	0.822	0.218	0.026	0.015	-0.091	0.029
Netherlands	-0.281	0.014	0.021	0.027	-0.073	-2.01e-02
Latvia	0.277	0.029	0.009	0.045	-0.081	7.39e-05
Slovenia	-0.538	-0.034	0.0133	0.043	-0.072	-2.44e-02
Greece	-0.932	0.02	0.017	0.051	-0.092	-1.34e-02
Ireland	0.550	-0.532	0.019	0.042	-0.107	-1.49e-02
Cyprus	0.096	-0.011	0.012	0.048	-0.083	-2.23e-03
Estonia	-0.247	-0.027	0.016	0.04	-0.079	3.93e-05

Table 3. Random effects of the population model for the **Health Monitoring** considered as Policy Intensity Index

Fixed Effects $HR(t) \sim$

**Parameter**	**value**	**SE**	**p-value**
$Intercept$	0.007	0.332	0.950
$I(t-1)$	0.771	0.207	~ 0
$newCases(t-1)$	-0.037	0.035	0.044
$admHosp(t-1)$	-0.019	0.150	0.754
$admICU(t-1)$	0.062	0.241	0.461
$deaths(t-1)$	-0.007	0.049	0.767

Table 4. Fixed effects of the population model for the **Health Resources** considered as Policy Intensity Index

**Random Effects** $HR(t) \sim$						
**ID**	**Intercept**	I_{t-1}	$newCases_{t-1}$	$admHosp_{t-1}$	$admICU_{t-1}$	$deaths_{t-1}$
Spain	0.143	0.172	-0.049	-0.337	0.571	0.084
France	0.059	-0.076	-0.027	-0.047	0.101	-0.036
Netherlands	0.155	0.073	0.021	0.070	-0.051	-0.037
Latvia	0.578	0.219	-0.001	0.003	-0.043	0.007
Slovenia	-0.203	-0.420	0.023	0.014	-0.044	0.005
Greece	-0.614	0.115	0.004	0.041	-0.070	0.0001
Ireland	0.307	-0.127	0.019	0.122	-0.353	-0.028
Cyprus	-0.191	-0.005	0.011	0.053	-0.044	0.002
Estonia	-0.234	0.048	-0.003	0.079	-0.064	0.002

Table 5. Random effects of the population model for the **Health Resources** considered as Policy Intensity Index

**Fixed Effects** $MP(t) \sim$			
**Parameter**	**value**	**SE**	**p-value**
$Intercept$	0.008	0.528	0.966
$I(t-1)$	0.951	0.066	~ 0
$newCases(t-1)$	0.021	3.38e-06	0.283
$admHosp(t-1)$	0.075	1.44e-01	0.299
$admICU(t-1)$	-0.119	1.55e-01	0.076
$deaths(t-1)$	0.003	0	0.798

Table 6. Fixed effects of the population model for the **Mask Policies** considered as Policy Intensity Index

**Random Effects** $MP(t) \sim$						
**ID**	**Intercept**	I_{t-1}	$newCases_{t-1}$	$admHosp_{t-1}$	$admICU_{t-1}$	$deaths_{t-1}$
Spain	0.414	0.002	-2.52e-10	-0.092	0.075	0
France	0.997	0.014	-1.27e-10	-0.068	0.062	0
Netherlands	0.205	-0.091	5.07e-10	0.089	0.029	0
Latvia	-0.110	0.092	1.28e-10	0.289	-0.359	0
Slovenia	-0.851	-0.001	-3.92e-11	-0.021	0.048	0
Greece	-0.684	-0.0003	-2.14e-11	-0.016	0.026	0
Ireland	0.225	-0.010	-8.18e-11	-0.077	-0.011	0
Cyprus	-0.002	-0.001	-5.13e-11	-0.023	0.035	0
Estonia	-0.194	-0.004	-6.29e-11	-0.079	0.093	0

Table 7. Random effects of the population model for the **Mask Policies** considered as Policy Intensity Index

**Fixed Effects** $SR(t) \sim$			
**Parameter**	**value**	**SE**	**p-value**
$Intercept$	0.009	0.645	0.965
$I(t-1)$	0.765	0.139	~ 0
$newCases(t-1)$	-0.006	0	0.739
$admHosp(t-1)$	0.002	0	0.954
$admICU(t-1)$	-0.001	0	0.973
$deaths(t-1)$	0.004	6.44e-06	0.735

Table 8. Fixed effects of the population model for the **School Restrictions** considered as Policy Intensity Index

**Random Effects** $SR(t) \sim$						
**ID**	**Intercept**	I_{t-1}	$newCases_{t-1}$	$admHosp_{t-1}$	$admICU_{t-1}$	$deaths_{t-1}$
Spain	0.531	1.70e-01	0	0	0	2.29e-09
France	0.754	4.50e-02	0	0	0	-1.01e-09
Netherlands	0.784	9.47e-02	0	0	0	2.16e-10
Latvia	0.367	-1.53e-01	0	0	0	9.015e-11
Slovenia	-1.027	-1.35e-01	0	0	0	-1.68e-09
Greece	-0.784	1.07e-01	0	0	0	-3.32e-11
Ireland	0.211	4.17e-02	0	0	0	1.30e-10
Cyprus	-0.239	-8.41e-05	0	0	0	1.41e-11
Estonia	-0.597	-1.70e-01	0	0	0	-1.50e-11

Table 9. Random effects of the population model for the **School Restrictions** considered as Policy Intensity Index

**Fixed Effects** $SD(t) \sim$			
**Parameter**	**value**	**SE**	**p-value**
$Intercept$	-0.007	0.754	0.977
$I(t-1)$	0.787	0.161	~ 0
$newCases(t-1)$	-0.018	0	0.178
$admHosp(t-1)$	0.059	0	0.035
$admICU(t-1)$	-0.012	0	0.653
$deaths(t-1)$	-0.071	0.083	0.040

Table 10. Fixed effects of the population model for the **Social Distancing** considered as Policy Intensity Index

**Random Effects** $SD(t) \sim$						
**ID**	**Intercept**	I_{t-1}	$newCases_{t-1}$	$admHosp_{t-1}$	$admICU_{t-1}$	$deaths_{t-1}$
Spain	0.564	0.168	0	0	0	0.078
France	0.866	-0.180	0	0	0	0.051
Netherlands	0.366	0.157	0	0	0	-0.049
Latvia	0.106	0.147	0	0	0	-0.027
Slovenia	-0.786	-0.078	0	0	0	0.063
Greece	-1.507	0.009	0	0	0	0.018
Ireland	0.312	-0.036	0	0	0	-0.169
Cyprus	-0.634	0.044	0	0	0	0.025
Estonia	0.712	-0.231	0	0	0	0.009

Table 11. Random effects of the population model for the **Social Distancing** considered as Policy Intensity Index

**Fixed Effects** $BR(t) \sim$			
**Parameter**	**value**	**SE**	**p-value**
$Intercept$	0.033	0.536	0.855
$I(t-1)$	0.712	0.083	0.000
$newCases(t-1)$	-0.036	0.019	0.060
$admHosp(t-1)$	-0.011	0.051	0.836
$admICU(t-1)$	0.115	0.051	0.024
$deaths(t-1)$	-0.121	0.042	0.004

Table 12. Fixed effects of the population model for the Business Restriction considered as Policy Intensity Index

Random Effects $BR(t) \sim$

**ID**	**Intercept**	I_{t-1}	$newCases_{t-1}$	$admHosp_{t-1}$	$admICU_{t-1}$	$deaths_{t-1}$
Spain	0.003	0.196	0	0	0.065	0.066
France	-0.052	-0.096	0	0	0.0382	-0.108
Netherlands	-0.468	-0.054	0	0	0.080	-0.072
Latvia	0.773	-0.032	0	0	-0.025	0.007
Slovenia	0.190	-0.469	0	0	-0.057	0.109
Greece	-1.092	0.140	0	0	-0.0003	0.031
Ireland	0.619	0.182	0	0	-0.048	-0.064
Cyprus	-0.251	0.002	0	0	0.002	0.009
Estonia	0.280	0.133	0	0	-0.055	0.021

Table 13. Random effects of the population model for the Business Restriction considered as Policy Intensity Index
